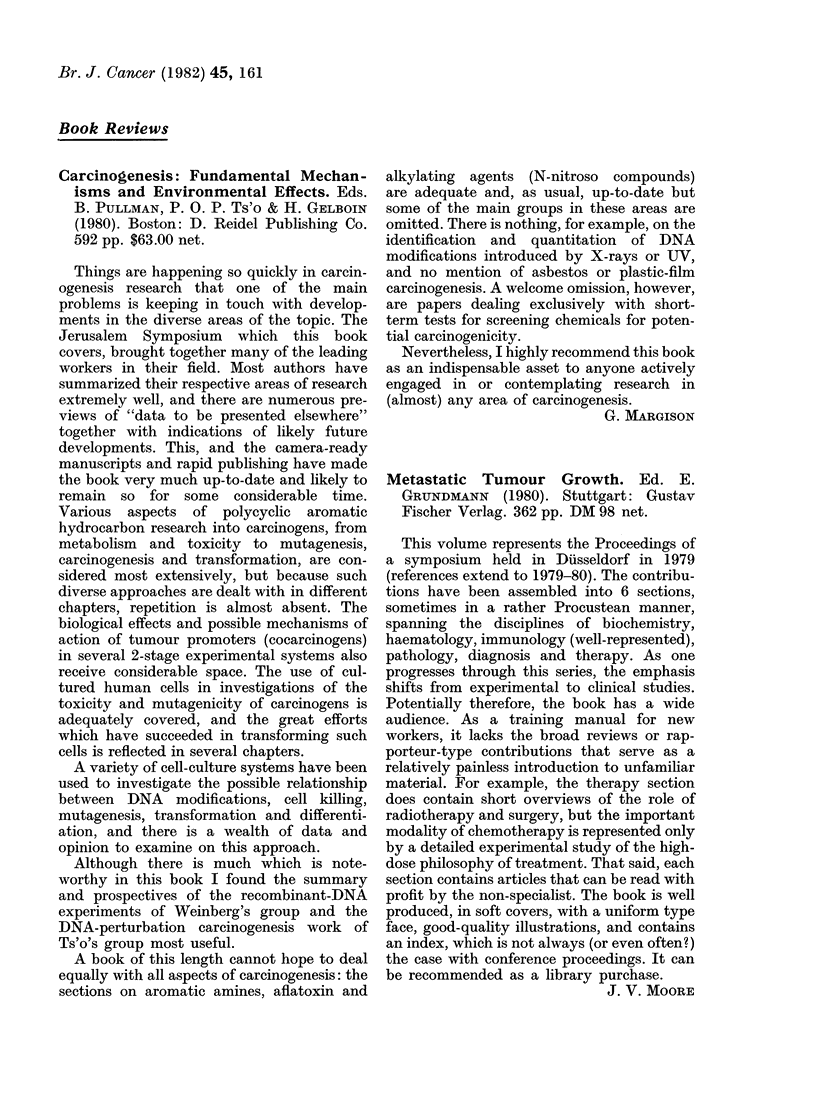# Carcinogenesis: Fundamental Mechanisms and Environmental Effects

**Published:** 1982-01

**Authors:** G. Margison


*Br. J. Cancer* (1982) **45**, 161

### *Book Reviews*

**Carcinogenesis: Fundamental Mechanisms and Environmental Effects.** Eds. B. PULLMAN, P. O. P. Ts'o & H. GELBOIN (1980). Boston: D. Reidel Publishing Co. 592 pp. $63.00 net.

Things are happening so quickly in carcinogenesis research that one of the main problems is keeping in touch with developments in the diverse areas of the topic. The Jerusalem Symposium which this book covers, brought together many of the leading workers in their field. Most authors have summarized their respective areas of research extremely well, and there are numerous previews of "data to be presented elsewhere" together with indications of likely future developments. This, and the camera-ready manuscripts and rapid publishing have made the book very much up-to-date and likely to remain so for some considerable time. Various aspects of polycyclic aromatic hydrocarbon research into carcinogens, from metabolism and toxicity to mutagenesis, carcinogenesis and transformation, are considered most extensively, but because such diverse approaches are dealt with in different chapters, repetition is almost absent. The biological effects and possible mechanisms of action of tumour promoters (cocarcinogens) in several 2-stage experimental systems also receive considerable space. The use of cultured human cells in investigations of the toxicity and mutagenicity of carcinogens is adequately covered, and the great efforts which have succeeded in transforming such cells is reflected in several chapters.

A variety of cell-culture systems have been used to investigate the possible relationship between DNA modifications, cell killing, mutagenesis, transformation and differentiation, and there is a wealth of data and opinion to examine on this approach.

Although there is much which is noteworthy in this book I found the summary and prospectives of the recombinant-DNA experiments of Weinberg's group and the DNA-perturbation carcinogenesis work of Ts'o's group most useful.

A book of this length cannot hope to deal equally with all aspects of carcinogenesis: the sections on aromatic amines, aflatoxin and alkylating agents (N-nitroso compounds) are adequate and, as usual, up-to-date but some of the main groups in these areas are omitted. There is nothing, for example, on the identification and quantitation of DNA modifications introduced by X-rays or UV, and no mention of asbestos or plastic-film carcinogenesis. A welcome omission, however, are papers dealing exclusively with short-term tests for screening chemicals for potential carcinogenicity.

Nevertheless, I highly recommend this book as an indispensable asset to anyone actively engaged in or contemplating research in (almost) any area of carcinogenesis.

G. MARGISON